# Ultra-narrow Band Perfect Absorber and Its Application as Plasmonic Sensor in the Visible Region

**DOI:** 10.1186/s11671-017-2203-9

**Published:** 2017-06-26

**Authors:** Dong Wu, Ruifang Li, Yumin Liu, Zhongyuan Yu, Li Yu, Lei Chen, Chang Liu, Rui Ma, Han Ye

**Affiliations:** 1 State Key Laboratory of Information Photonics and Optical Communications, Beijing University of Posts and Telecommunications, Beijing, 100876 China; 2 School of Science, Beijing University of Post and Telecommunication, Beijing, 100876 China

**Keywords:** Metamaterial, Absorber, Ultra-narrowband, Plasmonics, Sensing, FDTD

## Abstract

We propose and numerically investigate a perfect ultra-narrowband absorber with an absorption bandwidth of only 1.82 nm and an absorption efficiency exceeding 95% in the visible region. We demonstrate that the perfect ultra-narrowband absorption is ascribed to the coupling effect induced by localized surface plasmon resonance. The influence of structural dimensions on the optical performance is also investigated, and the optimal structure is obtained with the extremely low reflectivity (0.001) of the resonance dip. The perfect absorber can be operated as a refractive index sensor with a sensitivity of around 425 nm/RIU and the figure of merit (FOM) reaching 233.5, which greatly improves the accuracy of the plasmonic sensors in visible region. Moreover, the corresponding figure of merit (FOM*) for this sensor is also calculated to describe the performance of the intensity change detection at a fixed frequency, which can be up to 1.4 × 10^5^. Due to the high sensing performance, the metamaterial structure has great potential in the biological binding, integrated photodetectors, chemical applications and so on.

## Background

In recent years, plasmonic metamaterials based on the localized surface plasmon resonance (LSPR) have obtained significant progresses due to their electromagnetic properties and promising applications such as the monopole resonators [1–8], the light transmission enhancement [9–13], and the plasmonic sensors [14–21]. For a metamaterial absorber, it is advantageous to enhance the electromagnetic wave absorption, while the intrinsic optical losses of metals need to be carefully considered in the design of other devices. The first perfect metamaterial absorber is proposed and demonstrated by Landy [22]. Thereafter, perfect metamaterial absorbers have been developed rapidly [23–31], which can be generally classified as narrowband absorbers and broadband absorbers according to their different application requirements. Generally, the broadband absorbers can be used in energy harvestor while the narrowband absorbers are used in sensor and monochromatic photodetectors.

In sensing applications, the plasmonic refractive index sensor based on narrowband absorbers have attracted much attention. To date, many different types of the plasmonic refractive index sensors working in infrared and terahertz region have been reported including hybrid microcavities [32, 33], nanodisks [34], network-type metasurface [24], metal grating [28], and so on [35–37]. Note that, compared with the plasmonic sensors working in infrared, terahertz, and microwave region, the sensors operating at visible band can realize a smaller periodicity of metamaterial structure, which can improve the utilization of these devices in many practical applications, such as chemistry and biology [38]. Unfortunately, the previously published plasmonic refractive index sensors in visible region generally have a comparatively low FOM, which will greatly hamper their further development and application. In theoretical studies, in 2015, Zhou et al. theoretically proposed a refractive index sensor in the visible region using the subwavelength metal grating structures with an *S* of 300 nm/RIU, but the FOM is only 2 [28]. Liu et al. designed a multispectral sensor with deep-subwavelength plasmonic nanocavities and demonstrated a FOM of 58 [34]. With the efforts made by Liu et al., a refractive index sensor with the minimum FWHM reaching 3 nm and a FOM of 68.57 was obtained via the plasmonic structure with network-type metasurface [24]. In experimental studies, in 2014, Emiko and Tetsu experimentally demonstrated a LSPR sensor based on single Au nanostar structure with an *S* of 665 nm/RIU and an FWHM of up to 40 nm [39]. Cho et al. reported an experimental demonstration of a plasmonic refractive index sensor with the *S* reaching 378 nm/RIU [40]. Both in theory and experiment, many researchers have made great efforts to improve the FOM of the refractive index sensor operating in visible region. However, it is still a great challenge to design a plasmonic refractive index sensor with a high FOM in visible region, which severely limits its applications.

For sensors, it is very meaningful to increase FOM. For example, in the biological field, a higher FOM of refractive index sensor means a stronger performance in molecule detection. The FOM of the sensor in this work can reach 233.5, which is far higher than that of the published plasmonic refractive index sensor in the visible region [24, 28, 34]. The plasmonic sensor is based on the metal-dielectric-metal (MDM) periodic structure. Then, the structure also can operate as a perfect ultra-narrowband plasmonic absorber with an absorption efficiency over 95% and a FWHM of only 1.82 nm in visible region. We also investigate the influences of structure dimensions and material parameters on the optical properties of the metamaterial. Furthermore, we demonstrate that, compared to the common MDM structures, the usage of triangular nanoribbons in the structure is helpful to improve absorption performance. And meanwhile, the absorption mechanisms is also investigated and analyzed in detail. Considering the fabrication of the proposed structure, the triangular nanoribbons can be manufactured by many methods, such as e-beam lithography [41], molding [42], and imprint lithography [43]. It is expected that our work would be a guidance for the design of a plasmonic sensor.

## Methods

**Fig. 1:**
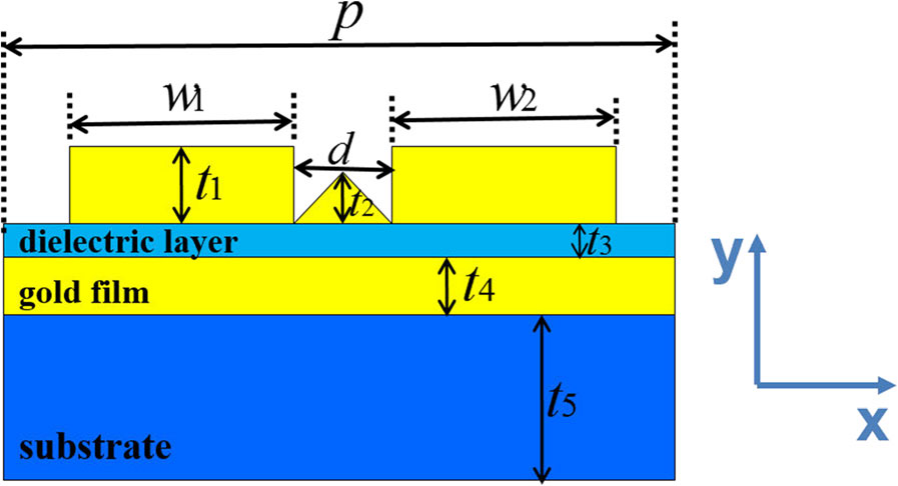
Schematic of the proposed metamaterial structure of one unit cell

Figure 1 illustrates the cross section of one unit cell for the proposed metamaterial structure. The structure consists of two gold nanoribbons array on a thin gold layer sandwiched between the dielectric layer and the substrate, and there is a triangular gold nanoribbon between the gold nanoribbons. In our simulation, the permittivity of gold is characterized by the Drude model. The dielectric of the middle layer and the substrate are set as NaF (*n* = 1.3) and MgF_2_ (*n* = 1.4), respectively. We use two-dimensional finite-difference time-domain (FDTD) method to calculate the transmission and reflection of the proposed structure and the absorption of the entire structure is defined as *A* = 1 − R − T. We set period boundary conditions in the x direction, and the transverse magnetic (TM) wave is incident normally onto the structure with polarization along the x direction.

**Fig. 2:**
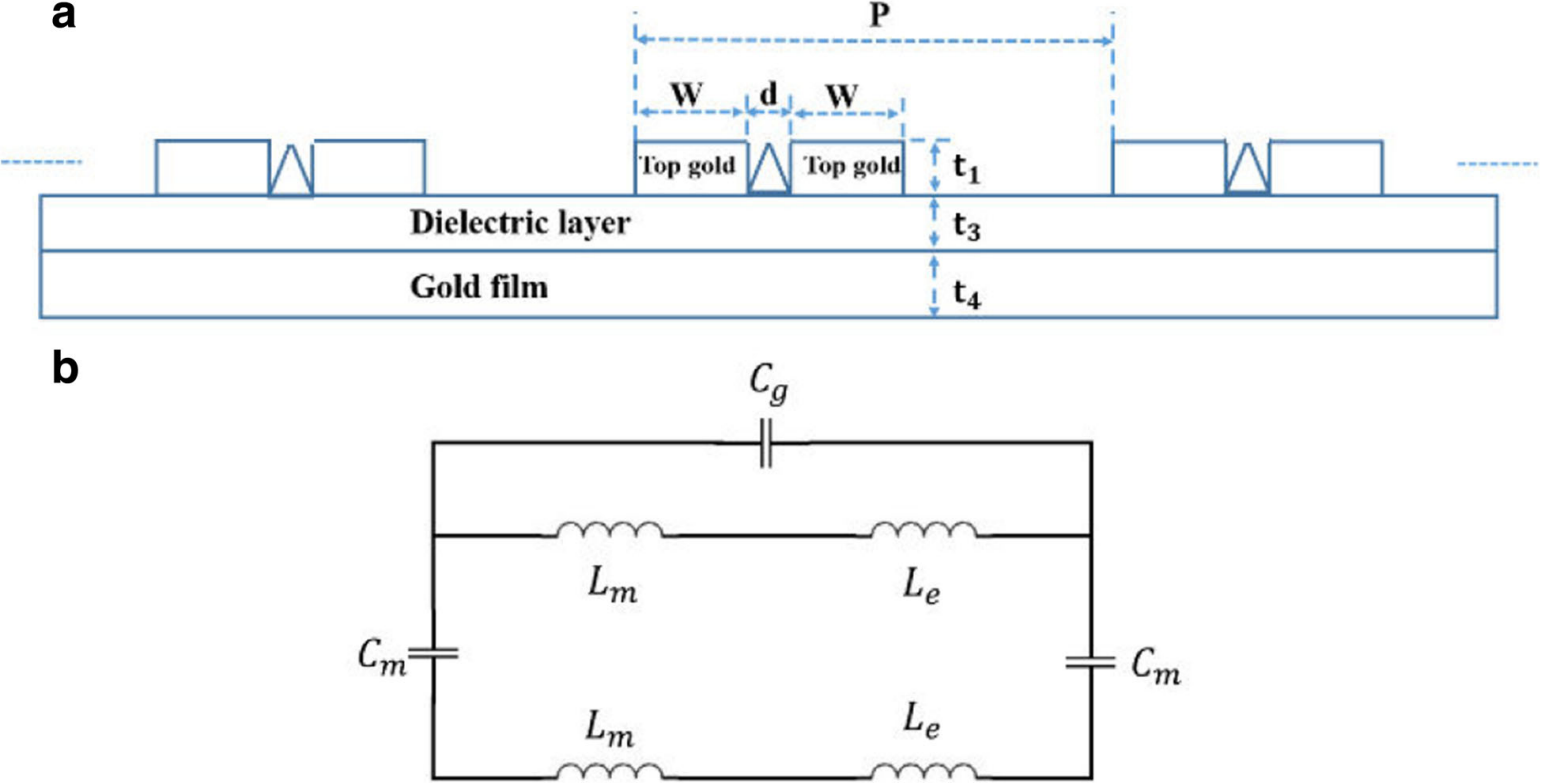
**a** Schematic of the metamaterial absorber structure. **b** Schematic of the equivalent LC circuit model for the structure of Fig. 6a

As we all know, the equivalent LC circuit model is widely used to qualitatively predict the magnetic resonance excited by LSPR for perfect absorber [44–46]. For the convenience of the discussion about LC model, the schematic of the metamaterial absorber structure is depicted in Fig. 2a. And the equivalent LC model is shown in Fig. 2b. Here, the gap capacitance between the nanoribbons in neighboring unit can be expressed as *C*_g_ = *ε*_0_ *t*_1_/(*P* − *d* − 2*w*), where *ε*_0_ is the dielectric permittivity of the surrounding environment. The capacitance *C*_*m*_ = *c*_1_ *ε*_3_ *ε*_0_(2*w* + *d*)/*t*_3_ is used to represent the capacitance between the nanoribbons and the gold film, where *c*_1_ is a coefficient owing to the non-uniform charge distribution on the surface of metal and *ε*_3_ is the permittivity of the dielectric layer [44–46]. The mutual inductance of the gold nanoribbons and the gold film is given by *L*_*m*_ = 0.5*μ*_0_(2*w* + *d*)*t*_3_, where *μ*_0_ is the permeability of the surrounding environment. To account for the contribution of the drifting charges in the gold nanoribbons and the gold film, the kinetic inductance is given by ${L}_e=\left(2 w+ d\right)/\left(\gamma {\varepsilon}_0{t}_1{\omega}_p^2\right)$, where *γ* is a coefficient accounting for the effective cross-sectional area of the gold nanoribbons and *ω*_p_ is the plasma frequency of the gold [44–46]. Then, the total impedance for the equivalent LC circuit model can be expressed as

1 $$ {Z}_{\mathrm{t} ot}=\frac{i\omega \left({L}_m+{L}_e\right)}{1-{\omega}^2{C}_g\left({L}_m+{L}_e\right)}-\frac{2 i}{\omega {C}_m}+ i\omega \left({L}_m+{L}_e\right) $$

The resonance wavelength can be obtained when *Z*_t*ot*_ = 0.

2 $$ {\lambda}_r=2\pi {c}_0{\left(\frac{C_m{C}_g\left({L}_m+{L}_e\right)}{C_m+{C}_g-\sqrt{C_m^2+{C}_g^2}}\right)}^{\frac{1}{2}} $$

The coupling between the nanoribbons in neighboring unit is very weak owing to the large gap (*P* − *d* − 2*w*) between the nanoribbons. The influence of *C*_*g*_ can be ignored when *C*_*g*_ is less than 5% of *C*_*m*_. Thus, in this situation, the resonance wavelength can be simplified to

3 $$ {\lambda}_r\approx 2\pi {c}_0\sqrt{\left({L}_m+{L}_e\right){C}_m} $$

where *L*_*m*_ = 0.5*μ*_0_(2*w* + *d*)*t*_3_, ${L}_e=\left(2 w+ d\right)/\left(\gamma {\varepsilon}_0{t}_1{\omega}_p^2\right)$, and *C*_*m*_ = *c*_1_ *ε*_3_ *ε*_0_(2*w* + *d*)/*t*_3_. In the LC circuit model, the influences of structural dimensions on the resonance wavelength can be qualitatively predicted by Eq. (3). It is easy to observe that the resonance wavelength *λ*_*r*_ would increase with larger permittivity (*ε*_3_) of the dielectric layer, owing to the increase of *C*_*m*_. Similarly, the larger width *w* will cause the larger values for *L*_*m*_, *L*_*e*_, and *C*_*m*_, resulting in a red shift of resonance wavelength. The increase of permittivity (*ε*_0_) of surrounding environment will result in larger *L*_*m*_ *C*_*m*_ values, while the other term *L*_*e*_ *C*_*m*_ is independent on the *ε*_0_ in Eq. (3). Thus, the resonance wavelength will increase with the increase of *ε*_0_.

## Results and Discussion

**Fig. 3:**
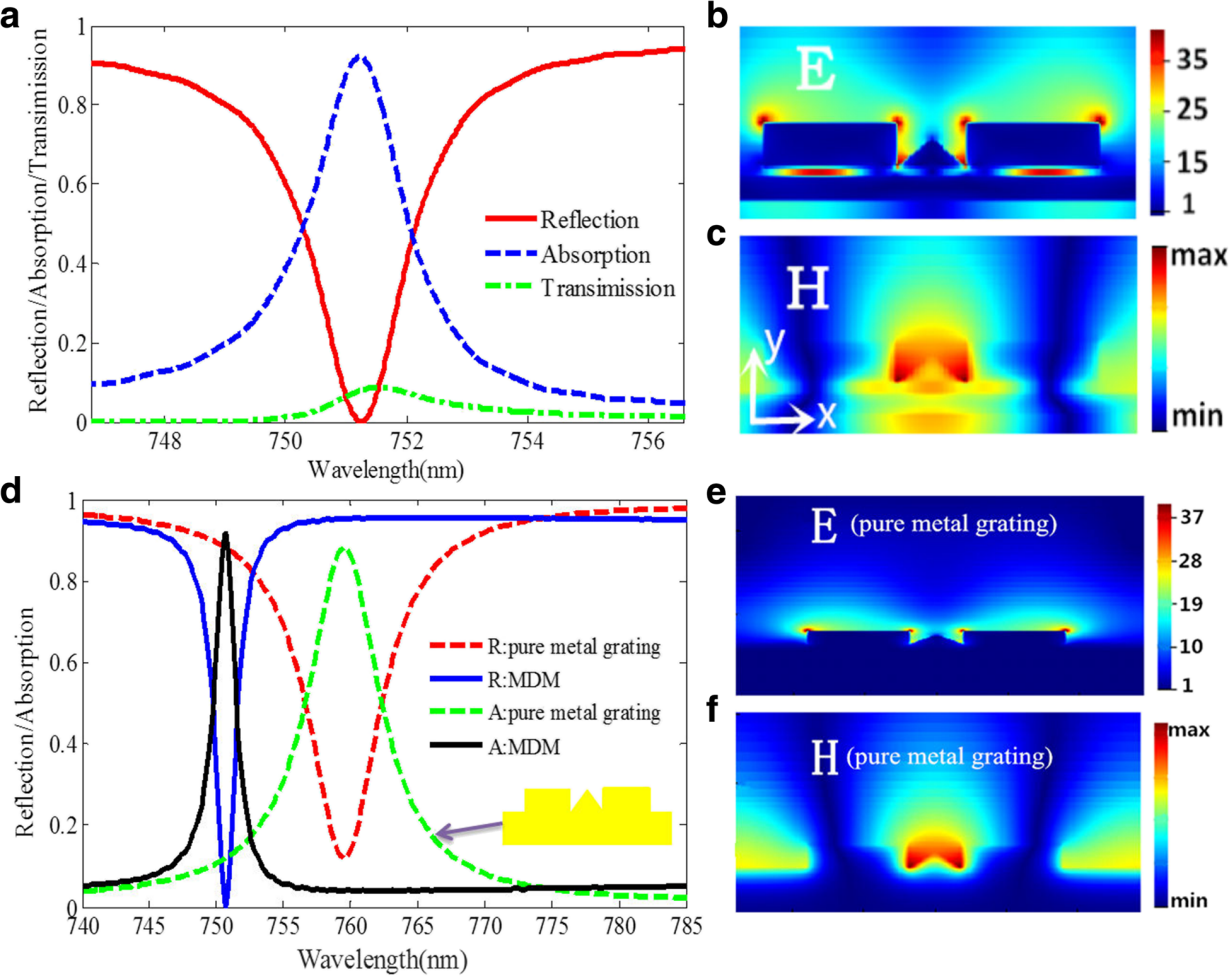
**a** Absorption, reflection, and transmission spectra of the proposed structure. **b** Distributions of the electric field *E* of the MDM structure at resonant peak. **c** Distributions of the magnetic field *H* of the MDM structure at resonant peak. **d** The reflection and absorption spectra of the MDM structure and pure metal grating structure. **e** Distributions of the electric field *E* of the pure metal grating structure at resonant peak. **f** Distributions of the magnetic field *H* of the pure metal grating structure at resonant peak

Then, we start the discussion with the following structure dimensions. The structure has a lattice period of *P* = 580 nm in x direction. The heights of the square gold nanoribbon and triangular gold nanoribbon are respectively set as *t*_1_ = 45 nm and *t*_2_ = 30 *nm*. The thicknesses of the dielectric layer, gold film, and substrate are *t*_3_ = 10 nm, *t*_4_ = 25 *nm*, and *t*_5_ = 165 *nm*, respectively. The width of the triangular gold nanoribbon and the square gold nanoribbon are *d* = 75 nm and *w*_1_ = *w*_2_ = *w* = 142*nm*, respectively. Figure 3a presents the simulated absorption, reflection, and transmission spectra of the designed structure. As shown in Fig. 3a, the absorption efficiency can reach up to 95%, and the reflectivity dip of the structure under 0.001 is found at 751.225 nm. The FWHM is 1.82 nm, which is much narrower than that of the previously reported narrowband absorber in the visible region [24, 28, 34, 39].

To elaborate the physical mechanism of the absorption peak, the distributions of the electric field E and the magnetic field H at the resonant peak are calculated and depicted in Fig. 3b, c. Clearly, as shown in Fig. 3b, the electric field amplitude in the gaps can reach a value as high as 35 times larger than the incident light. Hence, the proposed structure can realize not only the perfect absorption but also the electric field enhancement in a nanoslit, which is an important phenomenon in bio-sensing applications. As shown in Fig. 3c, the most magnetic field is concentrated at the space between two gold nanoribbons and some penetrates into the dielectric layer, which indicate the coupling effect resulting from the LSPR. Then, in order to further understand the influence of the dielectric layer and the gold film on the ultra-narrow FWHM and the high absorption performance, the absorption and reflection spectrum are analyzed and compared between the MDM structure and pure metal grating structure with the same dimensional parameters, as shown in Fig. 3d. Obviously, the MDM structure has a narrower FWHM and a lower reflectivity of the resonance dip. The electrical field and magnetic field of metal grating structure are simulated and presented in Fig. 3e, f, respectively. Obviously, compared with the magnetic field distribution of the MDM structure, the magnetic field of metal grating structure is only located on the surface of the triangular gold nanoribbon without the magnetic field passing through the metal, which can be used to explain the comparative result of the absorption between the MDM structure and the metal grating structure. Moreover, due to the coupling behavior in the structure, as shown in Fig. 3b, the electric field intensity between two gold nanoribbons and the thin gold film is about 40 times larger than that of the incident waves, which is much larger than that reported in ref. [25].

**Fig. 4:**
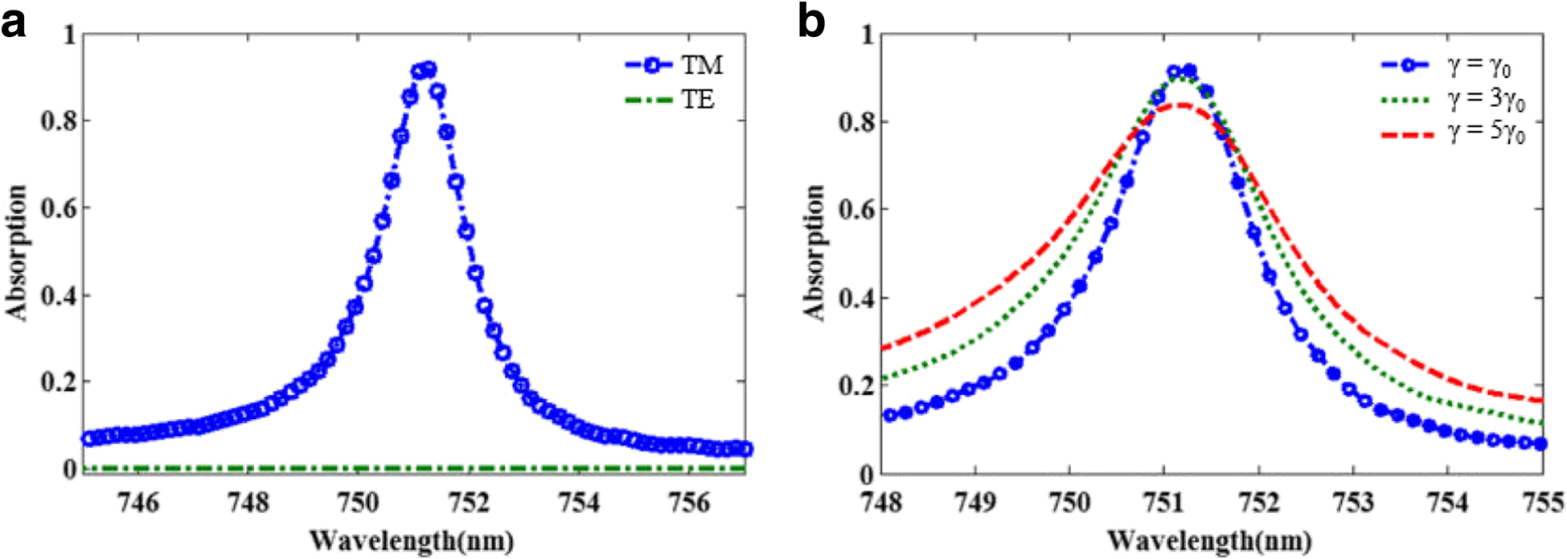
**a** The absorption spectra of the proposed structure under TE and TM polarization configurations. **b** Calculated absorption spectra in dependence on the damping constant of the gold film

Figure 4a shows the effect of polarization configuration of incident light on the absorption spectrum of the proposed metamaterial absorber. It can be seen that the structure has a sharp absorption peak in TM configuration, but not in TE configuration. Obviously, the LSPR cannot be excited by the incident light with TE configuration, which can be well explained by the asymmetrical structure of the absorber. Additionally, in an actual system, due to the surface scattering and grain boundary effects in the thin gold film, the damping constant of the thin gold film is likely higher than that of the bulk gold. To take into account the influence of the damping constant of the thin gold film, Fig. 4b shows the calculated absorption spectra of the damping constants of the gold film is three and five times higher that of the bulk gold. Obviously, absorption peaks with different amplitudes and FWHM are observed. The results show that the increased material loss of metal is unfavorable to further enhance the absorbing properties of the proposed narrowband absorber, which are consistent with the former research [17].

**Fig. 5:**
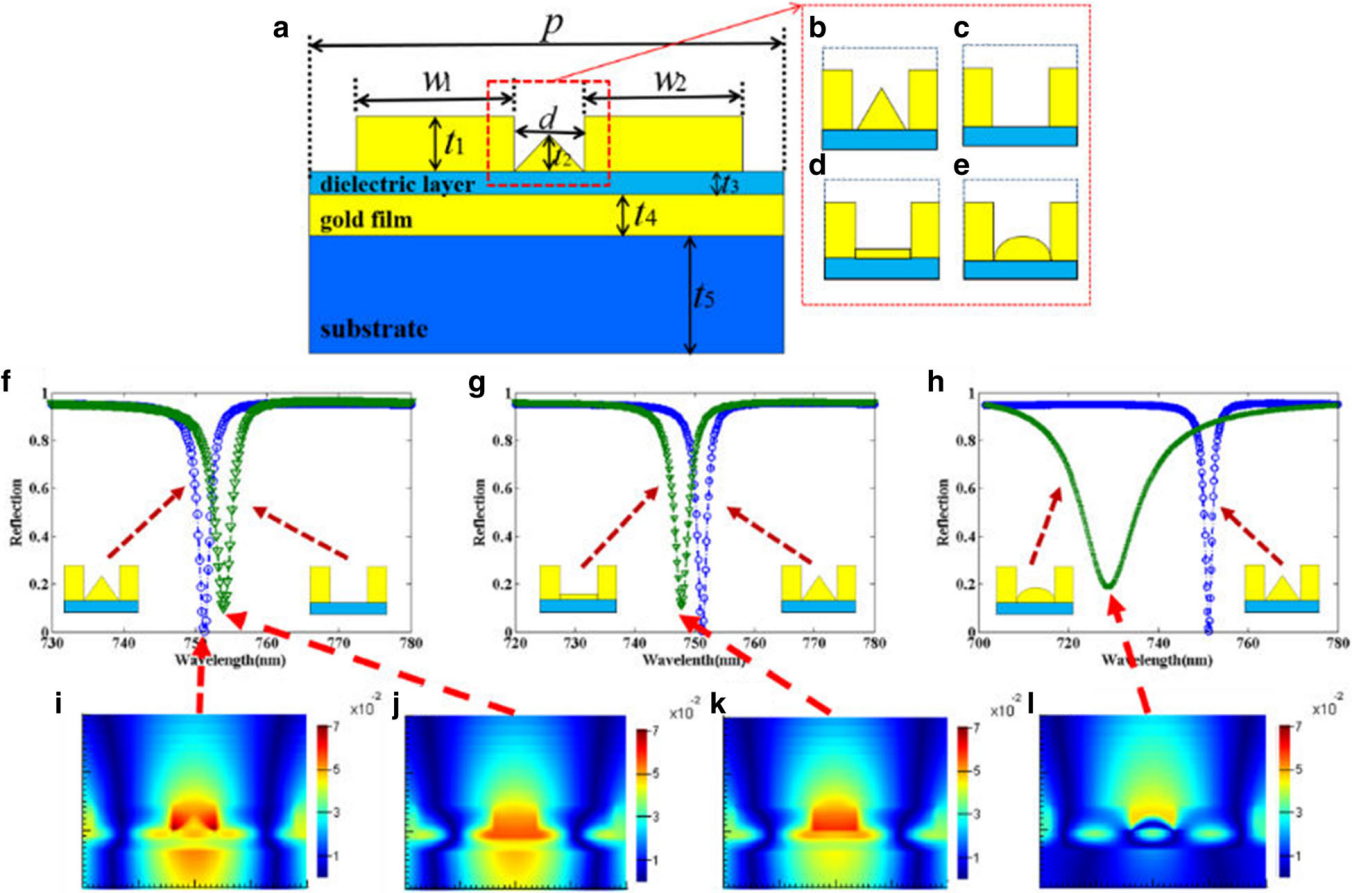
**a–e** Schematic of the proposed metamaterial with different nanostructures of one unit cell. **f–h** Reflection spectra of the different structures. **i–l** Distributions of the magnetic field H at the resonant peak of the corresponding structures

It is generally known that the properties of the metamaterial absorber are strongly influenced by the geometric shape and structural dimensions of the structure. Firstly, we investigate the effect of the triangular gold nanoribbon on the reflectance spectrum of the designed structure. The triangular gold nanoribbon of the structure is removed or changed into a square and semi-ellipse gold nanoribbon, respectively, as shown in Fig. 5c–e, with the other parameters kept unchanged in simulation. The reflection spectra of these three structures are analyzed and compared with that of the original structure as shown in Fig. 5f–h, respectively. It is easy to observe that the original structure can achieve a narrower FWHM and a lower reflectivity dip than three other structures. To better understand these results, as shown in Fig. 5i–l, the magnetic field (H) distribution at resonant peak of these four structures are respectively plotted and the color presents the intensity of the magnetic field. The magnetic field intensity of the original structure is obviously stronger than the three other structures. This means that LSPR can be excited more efficiently in the original structure, which results in a narrower FWHM and a lower reflectivity dip.

**Fig. 6:**
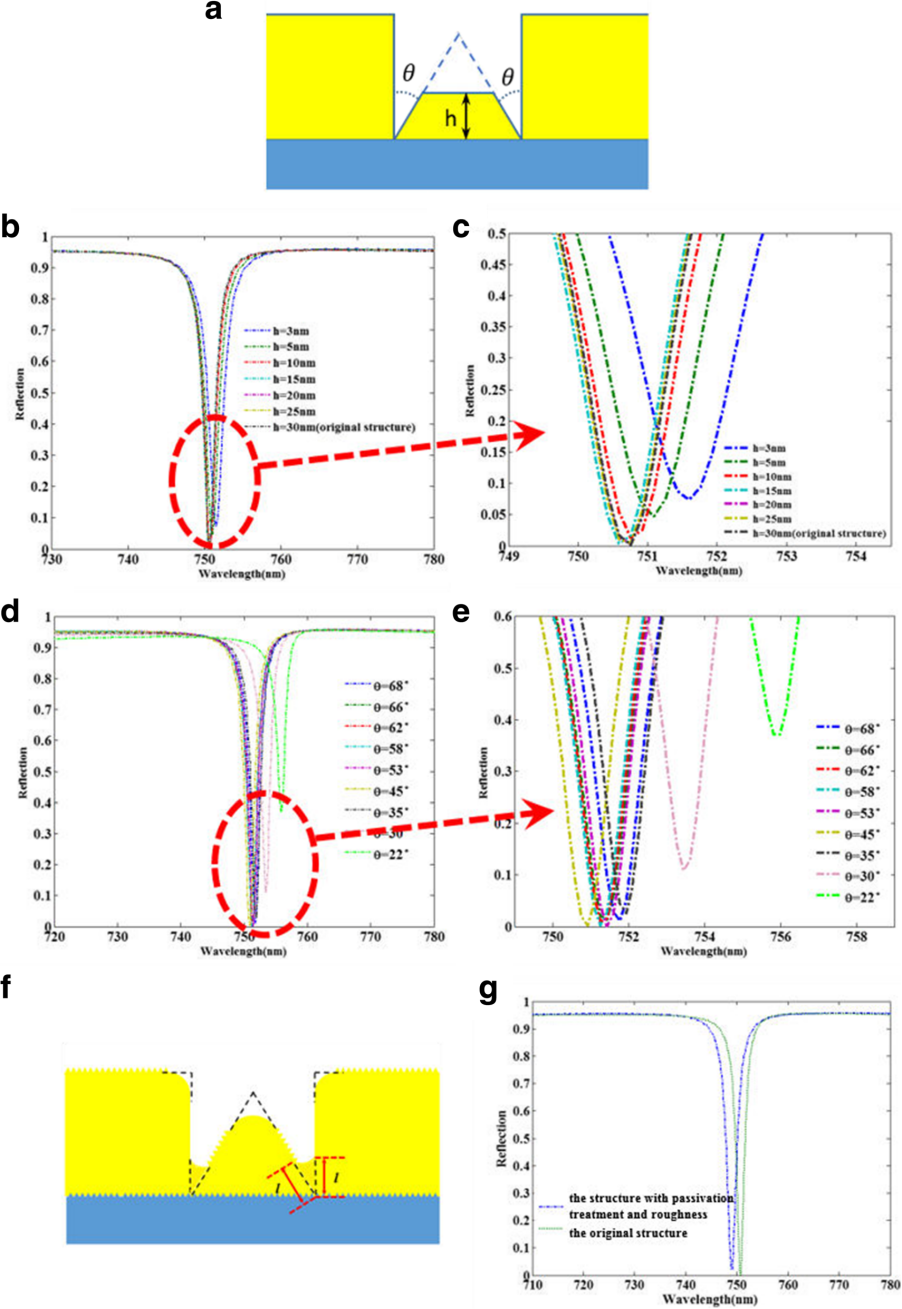
**a** The modified structure containing a trapezoidal nanoribbon with a same angle *θ* to the triangular nanoribbon. **b**, **c** Comparison of reflection spectra between the nanostructures with different heights *h*, when the *θ* keep unchanged. **d**, **e** Comparison of reflection spectra between the nanostructures with different angles *θ*, when the height *h* = 15 nm. **f** The modified structure with the roughness of the gold/dielectric surface and the passivation treatment for all sharp angles. **g** Comparison of reflection spectra between the modified structure and the original structure, when the *l* is set as 3 nm

From Fig. 5, the optical performance of the original structure with the usage of triangular nanoribbons is superior to that of the other structures. In order to make a further insight into the influences of the triangular nanoribbons on the optical performance, we give a detailed calculation and analysis for the modified structure shown in Fig. 6a, which contains a trapezoidal nanoribbon with a same angle *θ* to the triangular nanoribbon in original structure. Firstly, as shown in Fig. 6b, c, we investigate the optical performance of the modified structure dependence on different heights h of the trapezoidal nanoribbon when the angles *θ* remain unchanged. Obviously, when the height h is more than 10 nm, the optical performance of the structure will be kept almost unchanged, which shows the optical performance of the structure is robust in fabrication. As the height h is below 5 nm, the reflectivity dip increase, which can be explained that the height *h* is too small that would lower the effective area of the excitation of LSPR. As shown in Fig. 6d, e, we also investigate the optical performance of the modified structure dependence on different angles *θ* when the height h is set as 15 nm. It is easy to observe that the optical performance of the modified structure change little with the large angle range of 35° to 68°. However, the reflectivity dip increase obviously at the angle *θ* smaller than 30°, which can be understood that the too small angle *θ* may reduce the excitation efficiency of LSPR. Thus, by the detailed analysis to the influences of the different parameters of angles between the trapezoidal nanoribbon and the square nanoribbons on the optical performance, the perfect absorption performance of the original structure is attributed to the excitation of LSPR at the corner between the triangular nanoribbon and the square nanoribbons, which agrees well with the results of the magnetic field shown in Fig. 5i. At the same time, the structure can keep good optical performances in a large range of heights *h* and angles *θ*, which suggests a great relaxation to the fabrication robustness and makes the nanostructure become more realistic in experimental point of view. Finally, considering the fabrication processes of the actual nanostructure, Fig. 6f shows the geometry of the structure with the roughness of the gold/dielectric surface and the passivation treatment for all sharp angles. The comparison of the optical performance between the modified structure and the original structure are calculated and depicted in Fig. 6g. Obviously, the effect of the fabrication tolerance on the performance of the nanostructure is very small, which shows the robust optical performance in fabrication.

**Fig. 7:**
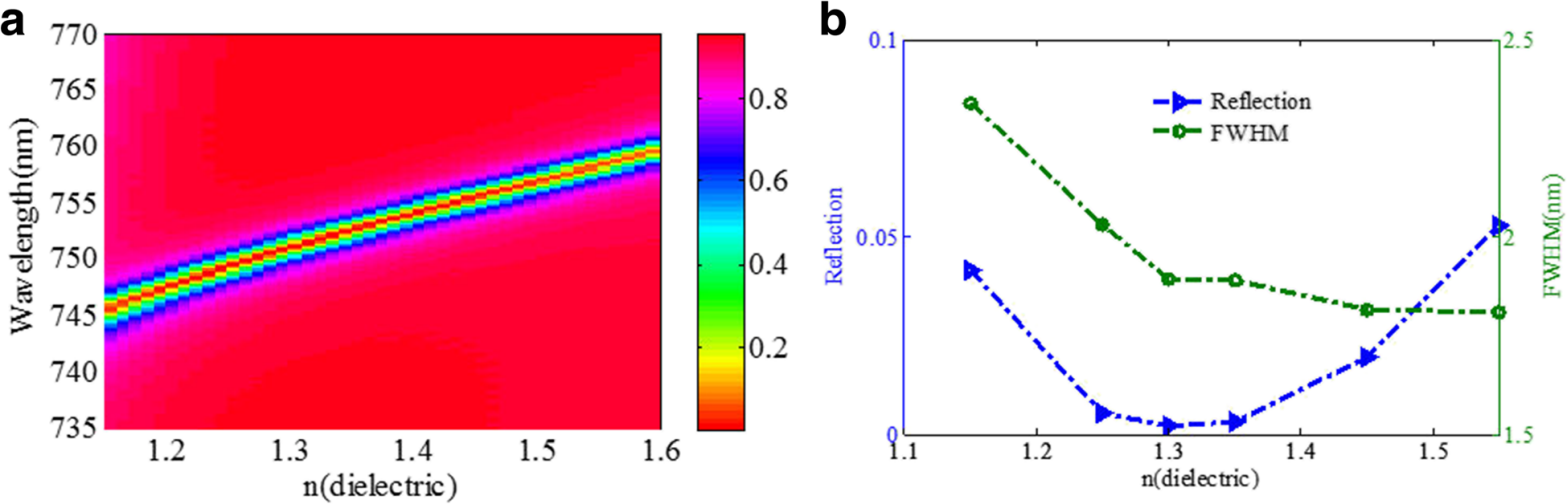
**a** Reflection spectra as a function of the refractive index of the dielectric layer. **b** Reflectivity of the resonance dip and FWHM as functions of the refractive index of the dielectric layer

Then, we also investigate the effects of the structure dimension and material parameters, by using FDTD method, on the reflectivity of dip, FWHM, and resonance wavelength of the designed structure. Several parameters will be studied including refractive index of the dielectric, gold nanoribbon width *w*, the gold nanoribbon width *d*, and the gold nanoribbon thickness *t*_1_. Figure 7 shows the effect of the refractive index of the dielectric layer on the reflectance spectrum of the metamaterial structure. As shown in Fig. 7a, the resonance wavelength redshifts obviously with increasing *n*_*dielectric*_, which is consistent with the prediction of the LC circuit model. As shown in Fig. 5b, the reflectivity dip is decreased firstly then increased when the *n*_*dielectric*_ is increased, whereas FWHM becomes narrower. The FWHM and reflectivity dip of the reflection spectrum depend strongly on the coupling strength between the nanoribbons and the gold film, resulting in the different optical performances with various dielectric materials of dielectric spacer between the nanoribbons and the gold film. The reflectivity dip is the minimum value when the refractive index of the dielectric layer is approximately 1.3. At the same time, the FWHM is around 1.85 nm, which is much narrower than that of the published narrowband absorber in the visible region [24, 28, 34, 39].

**Fig. 8:**
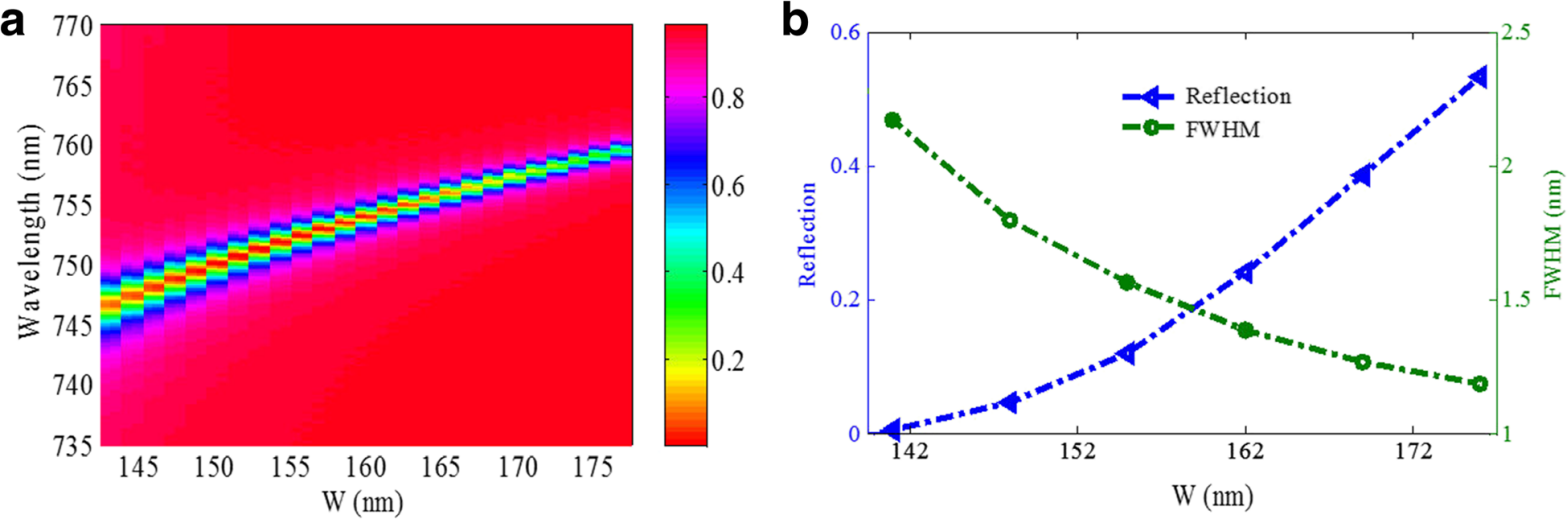
**a** Reflection spectra as a function of the gold nanoribbons width *w*. **b** Reflectivity dip and FWHM as functions of the gold nanoribbons width *w*

Figure 8 presents the influence of gold nanoribbon width *w* on the reflection spectrum of the metamaterial structure. As shown in Fig. 8a, when the gold nanoribbon width *w* changes from 140 to 177 nm, the resonant wavelength blueshifts, which agrees well with the results of the equivalent LC circuit model. Figure 8b shows that FWHM becomes narrower and the reflectivity dip increases with the increase of *w*. The increase of reflectivity dip may result from the increase of effective metal area for reflecting the incident light, with increasing *w*. The minimum values of reflectivity dip and the FWHM cannot be obtained simultaneously. However, in our design, both the values of the reflectivity dip and the FWHM change slightly in a wide range of *w* (140~162 nm), which is favorable to practical applications.

**Fig. 9:**
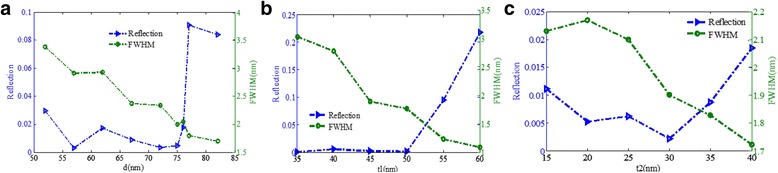
**a** Reflectivity dip and FWHM as functions of the triangular gold nanoribbon width *d*. **b** Reflectivity dip and FWHM as functions of the gold nanoribbons thickness *t*_1_. **c** Reflectivity of the resonance dip and FWHM as functions of the triangular gold height *t*_2_

Moreover, as shown in Fig. 9a, the reflectivity dip can sustain a lower value when the gold nanoribbon width *d* is between 55 and 75 nm while it increases obviously when *d* exceeds 76 nm, which can be explained that too large distance between the two nanoribbons may reduce the excitation efficiency for LSPR, thereby reducing the absorption efficiency of incident light. The FWHM becomes narrower with increasing *d*, and the optimum size of *d* is around 75 nm. From Fig. 9b, the reflectivity dip can keep a lower value when the gold nanoribbon thickness *t*_1_ changes from 35 to 50 nm while the FWHM becomes narrower. However, when *t*_1_ increases from 50 to 60 nm, the reflectivity dip increases obviously. We can understand the result like this, the nanoribbon is too thick that would increase the reflection of the incident light. Figure 9c shows that the minimum value of the resonance dip is obtained when the triangular gold height *t*_2_ is around 30 nm. In this structure, the reflectivity dip has been below 0.025 when the triangular gold height ranges from 15 to 40 nm, which is beneficial to design metamaterial structure owing to the excellent robustness performance.

**Fig. 10:**
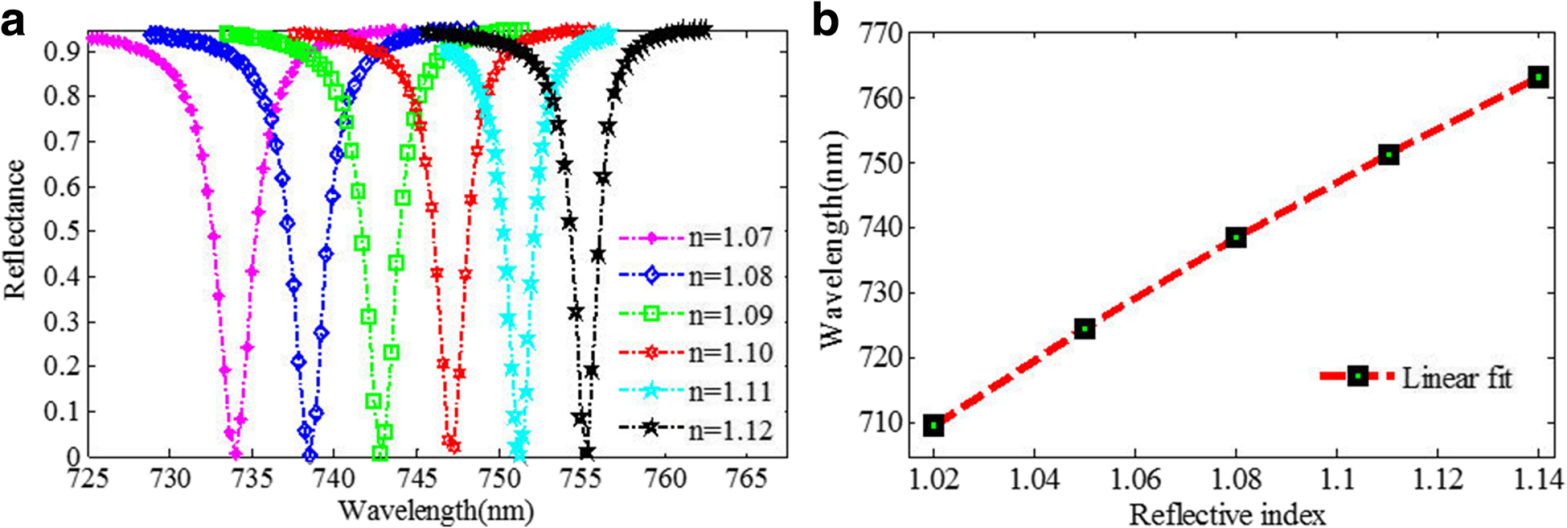
**a** Reflection spectra of the plasmonic refractive index sensor with various refractive index of the environment. **b** Resonant wavelength shift against the surrounding refractive index

It is generally known that the resonant wavelength of metamaterial structure is strongly dependent on the refractive index of the environmental medium, which has been widely used in sensing applications. Figure 10a shows that the resonant wavelength redshifts obviously when the refractive index of the environment increases, which is in agreement with the predication of the LC model, and the reflectivity dip can keep an extremely low value at the same time. When the RI increases from 1.07 to 1.12, the resonant wavelength shifts from 733.828 to 755.097 nm. The calculated wavelength sensitivity (*S*) is approximately 425 nm/RIU, and FWHM can be as narrow as 1.82 nm. Thus, the FOM can reach 233.5. As far as we know, the FOM is much higher than that of the previously published plasmonic refractive index sensor in visible region [24, 28, 34, 39]. The proposed plasmonic refractive index sensor shows good linearity, as shown in Fig. 10b.

**Fig. 11:**
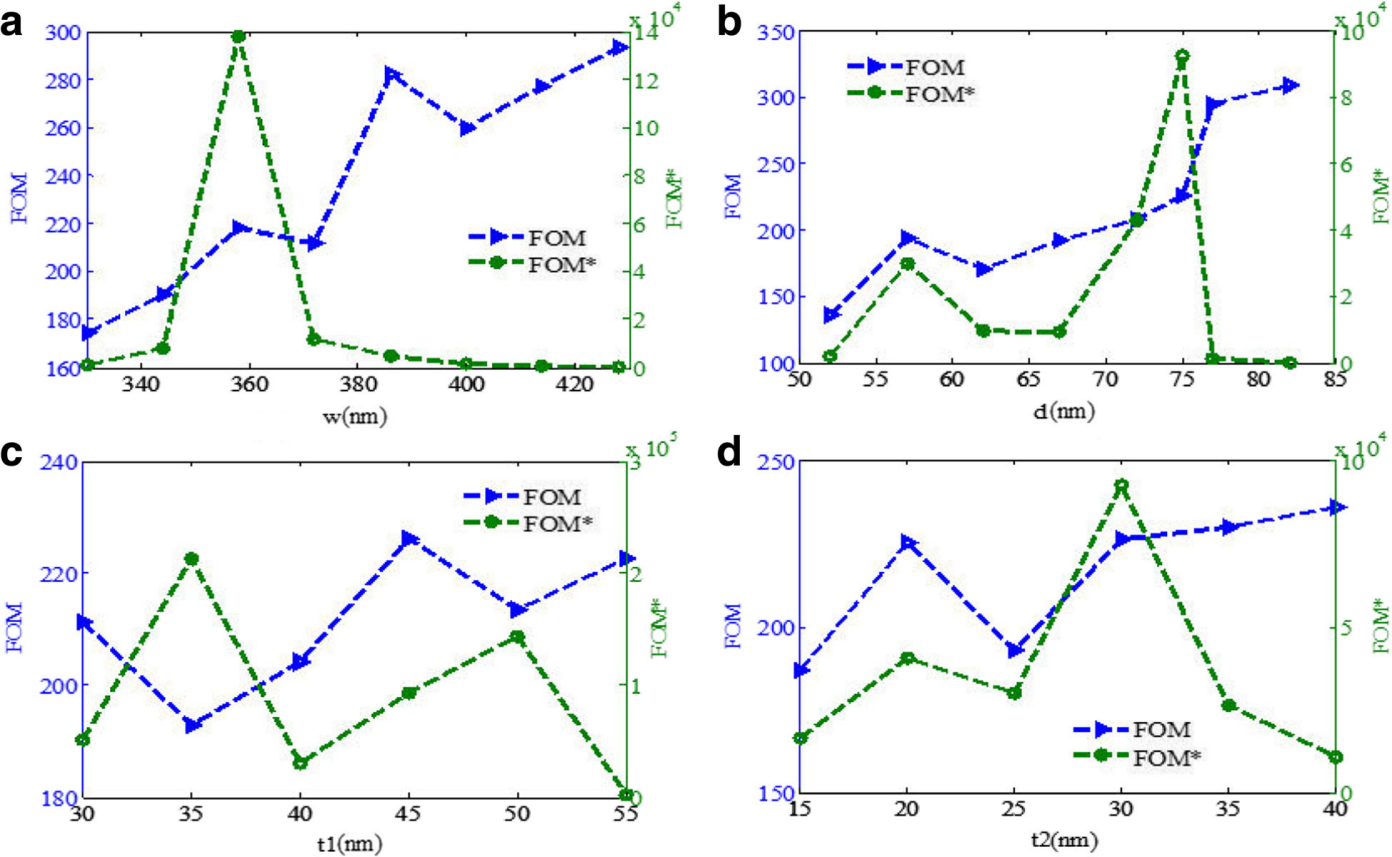
**a–d** FOM and FOM* as functions of the gold nanoribbon width *w*, the triangular gold nanoribbon width *d*, the gold nanoribbons thickness *t*_1_, and the triangular gold height *t*_2_, respectively

In practical applications, it is usually necessary to detect the relative intensity change at a fixed wavelength with the various refractive index of the surrounding medium, and the corresponding figure of merit is defined as FOM* = max |(*dI*/*dn*)/*I*| [17]. As shown in Fig. 11a, the FOM* changes obviously with decreasing *w*, and the maximum of FOM* can reach 1.4 × 10^5^ at the *w* of around 358 nm. Figure 11b shows that the FOM increases with decreasing *d* and a maximum of FOM* is obtained at *d* = 75 nm. As shown in Fig. 11c, when the gold nanoribbon thickness *t*_1_ is 35 nm, the FOM* is maximum. Moreover, Fig. 11d also shows that the maximum of FOM* is obtained when the triangular gold height *t*_2_ is about 30 nm. The characteristics of the FOM and FOM* with the changes of the structure dimensions is numerically investigated, which may offer certain guidance to design a high-performance plasmonic sensor.

## Conclusions

In summary, we propose and numerically demonstrate a nearly perfect ultra-narrow band absorber with an absorption reaching 95% in the visible region. We further make a detailed analysis in the influences of structural shape and structural dimensions on the optical properties of the metamaterial structure by using two-dimensional FDTD. Using the optimized structure dimensions, it presents the reflectivity dip as low as 0.001 with the FWHM of 1.82 nm at normal incidence in the visible region. In addition, we also demonstrated its sensing capability. Its sensitivity is around 425 nm/RIU and the FOM can reach 233.5. This is much better than that of the previously reported sensor in visible region [24, 28, 34, 39]. For its high sensing performance, the metamaterial structure may be found applications in the biological binding, integrated photodetectors, chemical applications, and so on.

## Abbreviations

FDTD: Finite-difference time-domain
FOM: Figure of merit
FWHM: Full width at half maximum
LSPR: Localized surface plasmon resonance
MDM: Metal-dielectric-metal
S: Sensitivity
TM: Transverse magnetic

## References

[1] Nielsen MG, Pors A, Albrektsen O, Bozhevolnyi SI (2012). Efficient absorption of visible radiation by gap plasmon resonators. Opt Express.

[2] Hedayati MK, Javaherirahim M, Mozooni B, Abdelaziz R, Tavassolizadeh A, Chakravadhanula VSK, Zaporojtchenko V, Strunkus T, Faupel F, Elbahri M (2011). Design of a perfect black absorber at visible frequencies using plasmonic metamaterials. Adv Mater.

[3] Dincer F, Karaaslan M, Sabah C (2015). Design and analysis of perfect metamaterial absorber in GHz and THz frequencies. Taylor & Francis.

[4] Lu H, Liu X, Mao D, Wang G (2012). Plasmonic nanosensor based on Fano resonance in waveguide-coupled resonators. Opt Lett.

[5] Ameling R, Langguth L, Hentschel M, Mesch M, Braun PV, Giessen H (2010). Cavity-enhanced localized plasmon resonance sensing. Appl Phys Lett.

[6] Jia KH, Zhang DW, Ma JS (2011). Sensitivity of guided mode resonance filter-based biosensor in visible and near infrared ranges. Sensors Actuators B Chem..

[7] Lee Joong W, Yang J-K, Sohn I-B (2014). Monopole resonators in planar plasmonic metamaterials. Optics Express.

[8] Kravets Vasyl G, Schedin F, Taylor S (2010). Plasmonic resonances in optomagnetic metamaterials based on double dot arrays. Optics Expres.

[9] Furkan D, Muharrem K, Sule C, Erkan T, Oguzhan A, MODERN PHYSICS LETTERS B, Altıntas O, Sabah C (2016). Multi-band polarization independent cylindrical metamaterial absorber and sensor application. Modern Physics Letters.

[10] Wang J, Zhou W, Li E-P (2009). Enhancing the light transmission of plasmonic metamaterials through polygonal aperture arrays. Optics Express.

[11] Farhang A, Anantha RS, Martin Olivier JF (2012). Compound resonance-induced coupling effects in composite plasmonic metamaterials. Optics Express.

[12] Frances J, Neipp C, Perez-Molina M, Belendez A (2010). Rigorous interference and diffraction analysis of diffractive optic elements using the finite-difference time-domain method. Comput Phys Commun.

[13] Jiang ZH, Lin L, Bossard JA, Werner DH (2013). Bifunctional plasmonic metamaterials enabled by subwavelength nano-notches for broadband, polarization-independent enhanced optical transmission and passive beam-steering. Optics Express.

[14] Li Y, Su L, Shou C, Yu C, Deng J, Fang Y (2013). Surface-enhanced molecular spectroscopy (SEMS) based on perfect-absorber metamaterials in the mid-infrared. Sci Rep.

[15] Jamali AA, Witzigmann B (2014). Plasmonic perfect absorbers for biosensing applications. Plasmonics.

[16] Sherry LJ, Chang SH, Schatz GC, Van Duyne RP, Wiley BJ, Xia Y (2005). Localized surface plasmon resonance spectroscopy of single silver nanocubes. Nano Lett.

[17] Liu N, Mesch M, Weiss T, Hentschel M, Giessen H (2010). Infrared perfect absorber and its application as plasmonic sensor. Nano Lett.

[18] Mayer KM, Hafner JH (2011). Localized surface plasmon resonance sensors. Chem Rev.

[19] Tamulevičius T, Gražulevičiūtė I, Urbonas D, Gabalis M, Petruškevičius R, Tamulevičius S (2014). Numerical and experimental analysis of optical response of sub-wavelength period structure in carbonaceous film for refractive index sensing. Opt Express.

[20] Lu X, Wan R, Zhang T (2015). Metal-dielectric-metal based narrow band absorber for sensing applications. Optics Express.

[21] Vasilantonakis N, Wurtz GA, Podolskiy VA (2015). Refractive index sensing with hyperbolic metamaterials: strategies for biosensing and nonlinearity enhancement. Optics Express.

[22] Landy NI, Sajuyigbe S, Mock JJ, Smith DR, Padilla WJ (2008). Perfect metamaterial absorber. Phys Rev Lett.

[23] Guanhai L, Xiaoshuang C, Oupeng L, Chengxue S, Yuan J, Lujun H, Bo N, Weida H, Wei L (2012). A novel plasmonic resonance sensor based on an infrared perfect absorber. J Phys D Appl Phys.

[24] Liu G, Yu M, Liu Z, Pan P, Liu X, Huang S, Wang Y (2016). Multi-band high refractive index susceptibility of plasmonic structures with network-type metasurface. Plasmonics.

[25] Xiaoyuan L, Lingxuan Z, Tongyi Z (2015). Nanoslit-microcavity-based narrow band absorber for sensing applications. Optics Express.

[26] Luo S, Zhao J, Zuo D, Wang X (2016). Perfect narrow band absorber for sensing applicationsn. Optics Express.

[27] Mandal P (2016). Plasmonic perfect absorber for refractive index sensing and SERS. Plasmonics.

[28] Wenchao Z, Kaiwei L, Chao S, Peng H, Mingbo C, Muxin Y, Yihui W (2015). Polarization-independent and omnidirectional nearly perfect absorber with ultra-thin 2D subwavelength metal grating in the visible region. Optics Express.

[29] Wu D, Liu Y, Yu L, Yu Z, Chen L, Li R, Ma R, Liu C, Zhang J, Ye H (2017). Plasmonic metamaterial for electromagnetically induced transparency analogue and ultra-high figure of merit sensor. Sci Rep.

[30] Peng Y, Jiang W, Eric A, Alexander G, Zhiming W (2016). Dual-band absorber for multispectral plasmon-enhanced infrared photodetection. J Phys D Appl Phys.

[31] Dong W, Chang L, Yumin L, Li Y, Zhongyuan Y, Lei C, Rui M, Han Y (2017). Numerical study of an ultra-broadband near-perfect solar absorber in the visible and near-infrared region. Optics Lett.

[32] Chowdhury Dibakar R, Su X, Zeng Y (2014). Excitation of dark plasmonic modes in symmetry broken terahertz metamaterials. Optics Express.

[33] Ralf A, Lutz L, Mario H, Martin M, Braun PV (2010). Cavity-enhanced localized plasmon resonance sensing. Appl Phys Lett.

[34] Liu Z, Shao H, Liu G, Liu X, Zhou H, Hu Y, Zhang X, Cai Z (2014). Gu Gλ3/20000 plasmonic nanocavities with multispectral ultra-narrowband absorption for high-quality sensing. Appl Phys Lett.

[35] Cheng F, Gao J, Stan L (2015). Aluminum plasmonic metamaterials for structural color printing. Optics Express.

[36] Huang Y-W, Chen Wei T, Wu Pin C (2012). Design of plasmonic toroidal metamaterials at optical frequencies. Optics Express.

[37] Huang X, Xiao S, Ye D (2010). Fractal, plasmonic metamaterials for subwavelength imaging. Optics Express.

[38] Zamarreno CR, Lopez S, Hernaez M, Del Villar I, Matias IR, Arregui FJ (2012). Resonance-based refractometric response of cladding-removed optical fibers with sputtered indium tin oxide coatings. Sensors Actuators B-Chemical.

[39] Kazuma E, Tatsuma T (2014). Localized surface plasmon resonance sensors based on wavelength-tunable spectral dips. Nanoscale.

[40] Cho SY, Briscoe JL, Hansen IA, Smith JK, Chang YM, Brener I (2014). Label-free plasmonic immunosensing for plasmodium in a whole blood lysate. IEEE Sensors J.

[41] Fujita T, Nishihara H, Koyama J (1982). Blazed gratings and Fresnel lenses fabricated by electron-beam lithography. Opt Lett.

[42] Xia Y, Kim E, Zhao XM, Rogers JA, Prentiss M, Whitesides GM (1996). Complex optical surfaces formed by replica molding against elastomeric masters. Science.

[43] Chang CH, Heilmann RK, Fleming RC, Carter J, Murphy E, Schattenburg ML, Bailey TC, Ekerdt JG, Frankel RD, Voisin R (2003). Fabrication of sawtooth diffraction gratings using nanoimprint lithography. J Vac Sci Technol B.

[44] Bai Y, Zhao L, Ju DQ, Jiang YY, Liu LH (2015). Wide-angle, polarization-independent and dual-band infrared perfect absorber based on L-shaped metamaterial. Optics Express.

[45] Feng R, Qiu J, Liu LH, Ding WQ, Chen LX (2014). Parallel LC circuit model for multi-band absorption and preliminary design of radiative cooling. Optics Express.

[46] Wang H, Wang LP (2013). Perfect selective metamaterial solar absorbers. Optics Express.

